# Atypical Sensory Processing in Neurodevelopmental Disorders: Clinical Phenotypes in Preschool-Aged Children

**DOI:** 10.3390/children11070875

**Published:** 2024-07-19

**Authors:** Federica Gigliotti, Federica Giovannone, Arianna Belli, Carla Sogos

**Affiliations:** Department of Human Neurosciences, Sapienza University of Rome, 00185 Rome, Italy; federica.gig@uniroma1.it (F.G.); federica.giovannone@uniroma1.it (F.G.); arianna.belli@uniroma1.it (A.B.)

**Keywords:** sensory processing, autism, neurodevelopmental disorders, preschool children

## Abstract

Background: Sensory processing issues are frequent in neurodevelopmental disorders (NDDs), with very variable prevalence rates ranging from 20% to 95%. This study aimed to investigate sensory processing in preschool-aged children with NDDs, to clarify the epidemiology, and to identify associated or correlated clinical and psychometric variables. Methods: A total of 141 NDD children (age range 2–5 years old) were included and enrolled in two subgroups: 72 with ASD and 69 with other NDDs. A standardized neuropsychological evaluation was assessed (Griffiths III/WPPSI-III/Leiter-R, ADOS-2) and the parents completed the CBCL ½–5, the SPM-P, and the ADI-R. Results: Atypical sensory processing was reported in 39.7% of the total sample, more frequently in ASD (44.4%) than in other NDDs (34.8%). No statistically significant differences were found regarding gender and developmental level. A positive correlation was found between sensory processing abnormalities and behavioral problems (*p* < 0.01). Conclusions: Compared to other NDDs, ASDs more frequently have atypical sensory processing and appear to present a specific vulnerability in the processing of proprioceptive and vestibular inputs. Our results suggest that sensory processing difficulties should be considered regardless of developmental level and in children with behavioral problems.

## 1. Introduction

### 1.1. Sensory Processing

The sensory system consists of eight modalities: vision, hearing, touch, taste, smell, vestibular system, proprioceptive system, and interoception system (i.e., the perception of internal sensations such as pain, temperature, itch, thirst, hunger, etc.). Sensory processing can be described as the ability to register, modulate, interpret, and organize sensory experiences to produce an adaptive behavioral response [1]. When sensory inputs are perceived and transmitted to the nervous system, sensory perception is easily influenced by brain states, such as sleep, wakefulness, or anesthesia. To accurately understand the environment, bottom-up sensory signals based on locomotion are interpreted by the brain together with prior experiences or emotional states, which is called top-down modulation. Therefore, sensory processing goes beyond the perception of internal or external inputs, involving cognitive phenomena to create an appropriate response to stimuli [2]. The cortical state plays a pivotal role in determining behavioral reactions to incoming sensory experiences. Neural activity in the electroencephalographic alpha band (8–12 Hz) has been linked to attentional and perceptual processing. For example, spontaneous alpha power detects briefly presented visual and tactile stimuli [3,4]. Considering these and other findings, several studies have outlined how alpha activity may play an active role in modulating sensory processing. It has been proposed that the brain may use the alpha rhythm to appropriately alternate between microstates of excitation and/or inhibition to be optimally ready to process or inhibit incoming information, executing a sensory control mechanism through the inhibitory activity of GABAergic interneurons [5,6]. These theories suggest that alpha oscillatory activity is critical in determining the perception and reactions to incoming sensory information [7]. According to some researchers, sensory processing arises from the interaction between the neurological threshold and self-regulation [8]. The neurological threshold is the amount of sensory stimuli needed by a person to notice them and to respond to them, while self-regulation is the behavioral administration of the received sensory information. Since sensory processing is crucial in interactions with the environment, children’s development and behavior are strongly influenced by their patterns of sensory processing [9]. Sensory processing difficulties are reported by 3–16% of the normal population [10,11,12], and they are much more frequent in individuals with neurodevelopmental disorders (NDDs), with very variable prevalence rates ranging from 20% to 95% depending on the study methodology and sample characteristics [11,13,14].

Considering that sensory processing issues are not peculiar to a single NDD and can also be present in TD children, some authors proposed the existence of a specific condition called sensory processing disorder (SPD) [15,16]. A nosology for SPD proposed by experts considers this disorder as having three main categories: sensory modulation disorder (SMD), sensory discrimination disorder (SDD), and sensory-based motor disorder (SBMD) [15]. SMD includes three subtypes: sensory over-responsivity (SOR), sensory under-responsivity (SUR), and sensory craving (SC). Sensory modulation refers to the ability of the individual to regulate sensory stimuli and produce appropriate reactions. Atypical behaviors associated with SOR are exaggerated and prolonged responses to sensory input considered low intensity by most children. Atypical behaviors with SUR include an absent/slowed responses to sensory information or the need for extremely strong sensory stimulation to elicit an awareness of the stimuli. Finally, atypical behaviors related to SC include searching for more or stronger sensory stimulation than usual [17]. SDD refers to difficulties with the ability to accurately interpret specific features of sensory stimuli, such as the duration, intensity, or spatiotemporal characteristics. Finally, SBMD is usually present in the case of abnormal tactile, proprioceptive, or vestibular processing and includes two phenotypes: postural disorder, which encompasses problems with balance, co-ordination, and body stabilization during movement or at rest; and dyspraxia, which is defined as an impairment in motor planning and sequencing movement [18].

However, only one of the SPD subtypes (i.e., SMD) is acknowledged by the Diagnostic Manual for Infancy and Early Childhood [19] and the Zero to Three Diagnostic Classification of Mental Health and Developmental Disorders of Infancy and Early Childhood: Revised edition [20], and SPD is not included in DSM-5 or ICD. In 2012, the American Academy of Pediatrics recommended that, generally, SPD should not be diagnosed, given the lack of a universally accepted diagnostic framework [16].

### 1.2. Neurodevelopmental Disorders

Neurodevelopmental disorders (NDDs) are a broad group of conditions involving some form of disruption in brain development and causing the impairment of functioning [21,22]. The Diagnostic and Statistical Manual for Mental Disorders—Fifth Edition (DSM-5) gathers the following conditions into the diagnostic category of NDDs: intellectual disability (ID), attention deficit and hyperactivity disorder (ADHD), autism spectrum disorder (ASD), communication disorders (CDs, including specific language impairment), specific learning disorders (LDs), and motor disorders (including developmental co-ordination disorder, DCD, and tic disorders) [23]. One of the defining characteristics of these disorders is that they begin in childhood, before puberty, even if symptoms may not fully manifest until the demands for the affected functions exceed the child’s capacities. NDDs can also be distinguished from many other neuropsychiatric disorders by their clinical course: although their clinical manifestation may change across different developmental stages, they tend to persist into adulthood and show a steady course, rather than the remitting and relapsing pattern commonly found, for example, in schizophrenia and mood disorders [21]. Furthermore, the overlap between these disorders or their symptom dimensions is frequent [22]. Finally, these conditions share a male predominance, a high heritability, and some common risk factors, such as environmental pollution, late pregnancies, and preterm birth [24,25,26]. Nevertheless, NDDs are highly heterogeneous in their clinical characteristics, etiologies, treatment responses, and prognoses. A recent systematic review on the prevalence of NDDs [27] reports worldwide prevalence rates that facilitate an understanding of why these conditions can be considered one of the current global public health challenges: ADHD, 5–11%; ASD, 0.7–3%; CD, 1–3.42%; ID, 0.63%; LD, 3–10%; and MD, 0.76–17%. The early detection of these disorders is crucial in order to increase the effectiveness of remediation programs and obtain better outcomes for affected individuals [28].

### 1.3. Atypical Sensory Processing in NDD

The research has shown an interest in understanding atypical sensory processing in NDDs as early as 1963 when occupational therapist and psychologist J.A. Ayres conducted some of the first works of research evaluating sensory issues in individuals with diverse developmental abnormalities. She then theorized the concept of “sensory integration”, which consists of “the organization of sensations for use”, referring to the ability to produce adequate responses to sensory inputs [29]. A later crucial contribution to sensory processing research came from Dunn’s work, which led to one of the most recognized models for sensory disorders [30]. In the last decades, many studies have focused on sensory problems in children with ASD. However, interesting evidence regarding sensory processing is also available in many other NDDs. Studies on sensory processing in ASD children showed that up to 95% of them report hyper- and hypo-sensitivities in different sensory modalities [31]. Atypical sensory processing has indeed been identified as one of the core features of ASD since the first descriptions of affected individuals by Kanner and Asperger, and, since 2013, hyper- or hyporeactivity to sensory inputs or unusual interest in sensory aspects of the environment are included among the diagnostic criteria of ASD in DSM-5. Examples of these symptoms are an apparent indifference to pain or temperature, an adverse response to specific sounds or textures, the excessive smelling or touching of objects, and a visual fascination with lights or movement [23]. These phenomena, however, manifest differently across individuals, who can appear to be unaware of specific sensory stimuli (hypo-responsivity) or show distress and attempt to avoid sensory inputs (hyper-responsivity). Hypo- and hypersensitivity are possible even within a single individual (e.g., hypersensitive to touch and hypersensitive to sight) [32,33]. Historically, proximal senses such as smell, taste, and touch were considered to be particularly affected and to be a sign of developmental immaturity [34,35], and, more recently, the research has shown a growing interest in multisensory integration (MSI) in ASD [36]. Some authors identified distinct sensory processing subtypes in autism [32]. However, limited consensus exists regarding the pattern of sensory impairments in individuals with ASD [37].

Although there is a large amount of behavioral literature on sensory characteristics in ASD individuals, less is known about the neural networks involved in sensory processing in this clinical population. Functional magnetic resonance imaging (fMRI) studies showed that the presentation of simple auditory and visual stimuli resulted in an increased activation in primary sensory cortices and in limbic areas related to emotion processing and regulation in children with ASD, compared to controls. These findings suggest atypical features of sensory processing in ASD at both a lower level (i.e., primary or association sensory cortex) and a higher level (i.e., attentional or limbic cortices) [38]. In addition to that, a critical role in sensory processing in ASD appears to be played by the white matter. Increased tactile defensiveness has been shown to correlate with abnormal signals of the inferior longitudinal fasciculus in diffusion tensor imaging (DTI), a technology used to characterize the white matter microstructure in a sample of ASD children. This may reflect an aberrant connection between limbic structures in the temporal lobe and the inferior parietal cortex. Furthermore, data from the same sample led the authors to propose a role for the splenium of the corpus callosum in modulating attentional orienting, a process affecting the elaboration of sensory inputs [39]. Among children with ID, a higher incidence of sensory processing difficulties has been reported compared to TD children, even when ID is not part of a specific syndrome [40]. In addition, researchers found that sensory processing impairments in individuals with ID were related to various adaptive functions and changed across the lifespan [41]. Finally, although severe–profound ID is usually associated with more severe motor and maladaptive skills, it has also been reported that ID severity has a minimal impact on sensory processing abilities and that all levels of ID show similar patterns of sensory impairment. However, a significant difference between mild and severe–profound ID was found for auditory-sensation-seeking behaviors [41].

In specific language impairment, the altered auditory processing of speech sounds has been reported [42], and this sensory processing dysfunction may explain the poor phonological development, impaired word production, and difficulties in word comprehension that characterize this NDD [43]. Similar findings are reported in individuals with dyslexia [44]. In children with ADHD, parents reported more sensory processing problems than those found in TD peers in all sensory modalities [45]. In addition, tactile defensiveness, often present in children with ADHD, has been associated with anomalous central responses to a somatosensory stimulus related to disruptions in neural inhibition [46]. Furthermore, some researchers proposed abnormal tactile sensory processing at an early age as a marker of future attention dysfunctions, contributing to the early detection of NDD and, more specifically, ADHD [26]. Regarding children with DCD, it has been reported that most of them show some or definite differences in sensory processing compared to their TD peers [47], with worse performances in visual perception and somatosensory perceptual tasks [48]. It has also been proposed that sensory issues may contribute to the etiology and development of this neurodevelopmental condition [49]. In summary, sensory processing difficulties are a highly prevalent condition in children with NDDs; they affect the development of autonomy, basic and instrumental activities of daily living, play, social participation, and learning [50], thus having a strong impact on the quality of life and the developmental outcome of these children. In some cases, behaviors related to sensory processing dysfunctions (e.g., stereotypies) can lead families to seek medical attention and be a diagnostic cue for clinicians, leading to a prompt recognition and rehabilitation of neurodevelopmental conditions [51]. Furthermore, knowing the sensory characteristics of a child is often helpful in choosing appropriate and more effective rehabilitation therapy interventions [52].

### 1.4. Aim of the Study

The present study aims to evaluate the presence of atypical sensory processing in preschool-aged children with diverse neurodevelopmental conditions to clarify the epidemiology in terms of prevalence and gender differences. Additionally, the level of development, autistic symptoms, and behavioral problems were drawn to identify any clinical and psychometric variables associated with sensory processing difficulties. Furthermore, we compared the profiles of children with ASD and those with other NDDs to verify any differences between the two subpopulations that could have implications for diagnostic and therapeutic strategies.

## 2. Materials and Methods

### 2.1. Participants

The present study was conducted in the Neurodevelopmental Disorders Day Hospital of the Department of Neuroscience and Mental Health of Sapienza University of Rome. Participants were preschool children referred for diagnostic assessment for suspected NDD.

Inclusion criteria were: (a) an age between 2 and 5 years; (b) a diagnosis of one (or more) NDDs according to DSM-5 criteria (ASD, developmental delay, communication disorders, developmental co-ordination disorder, and ADHD). Exclusion criteria included not being able to complete a structured assessment of development or intelligence or having caregivers not sufficiently fluent in Italian to complete the entire assessment. Recruitment was performed from October 2021 to July 2023.

### 2.2. Procedure

The variables investigated were age, sex, level of development, core autistic symptoms, behavioral problems, and sensory processing.

The diagnosis in all subjects was clinically established by a highly skilled multidisciplinary team, comprising child neuropsychiatrists and rehabilitation professionals from the Department of Neuroscience and Mental Health of Sapienza University of Rome. The evaluation was conducted using a combination of history, observation, and standardized instruments.

#### 2.2.1. Level of Development

Mental development was assessed using standardized scales.

The Griffiths Scales of Child Development (3rd edition) were the primary assessment tool for most patients. This comprehensive developmental measure, applicable from birth to 6 years, evaluates five key areas (Foundations of Learning, Language and Communication, Eye and Hand Coordination, Personal–Social–Emotional, and Gross Motor), providing an overall measure of a child’s development (Developmental Quotient) [53,54].

For some patients, a cognitive scale was chosen instead of a developmental scale based on the child’s clinical features and attitudes. The Wechsler Preschool and Primary Scale of Intelligence—3rd edition (WPPSI-III) is an intelligence test designed for children aged 2 years 6 months to 7 years 7 months. It consists of 15 subtests, including core subtests that provide a Verbal Quotient, a Performance Quotient, a Processing Speed Quotient, and a Full-Scale Intelligence Quotient [55].

The Leiter International Performance Scale, Revised (Leiter-R) measures nonverbal intellectual functioning for individuals aged between 2 and 20 years and 11 months, administered without vocal instructions. It includes Visualization and Reasoning (VR) and Attention and Memory (AM) batteries. The VR battery’s subtests assess traditional intelligence constructs, such as nonverbal reasoning, visualization, and problem-solving, and are used to obtain Intelligence Quotient estimates [56].

Based on their Developmental Quotient (DQ) or Intelligence Quotient (IQ), participants were classified as belonging to one of three groups: (1) grossly abnormal development, for those with DQ or IQ smaller than 70; (2) mildly abnormal development, for those with DQ or IQ of 70–84; and (3) normal development, for those with DQ/IQ equal to or greater than 85.

#### 2.2.2. Autistic Symptoms

Autistic symptoms were assessed using standardized tools in patients for whom a diagnosis of ASD was clinically suspected.

The Autism Diagnostic Interview—Revised (ADI-R) is a structured interview composed of 93 questions that investigate three functional domains: Language and Communication (scale A), Reciprocal Social Interactions (scale B), and Restricted, Repetitive, and Stereotyped Behaviors and Interests (scale C). The examiner codes caregivers’ answers, and the results are then scored and interpreted using an age-dependent diagnostic algorithm. For each domain, a cut-off score indicates clinically relevant results. For our study, we gathered ADI-R scores for each scale as dichotomous variables (equal to or higher than cut-off/lower than cut-off) [57].

The Autism Diagnostic Observation Schedule-II (ADOS-II) is a standardized and semi-structured assessment of children’s communication, social interaction, play, imagination, and restricted and repetitive behaviors. During administration, the examiner interacts directly with the child in play and social activities. It has five modules with different activities to observe the behavior of participants with various developmental and linguistic levels. Each patient is assessed with only one module. For our study, modules Toddler 1 and 2 were used based on each child’s expressive language level and age. The clinician codes the behaviors observed during administration, and a selection of the scores is entered into the diagnostic algorithm, yielding a total score. All modules included two cut-off scores for the classification of “autism spectrum disorder” and “autism”, and the total score matches with a comparison score (also known as calibrated severity score, CSS), which indicates the severity of the autistic symptoms. Based on their CSS, participants were grouped into three categories: (1) moderate–severe autistic symptoms for those with CSS of 5 to 10 (or classified as “at moderate–severe risk” in module Toddler); (2) mild autistic symptoms when CSS was 3 or 4 (or classified as “at mild–moderate risk” in module Toddler); and (3) minimal or absent autistic symptoms for those with a CSS of 1 or 2 (or classified as “little or no risk” in module Toddler) and for those who did not raise clinical suspicion for ASD [58].

#### 2.2.3. Behavioral Problems

The Child Behavior Checklist for ages 1½–5 years was used to investigate problem behaviors. This 100-item parent-report questionnaire was designed to record emotional and behavioral problems in preschoolers. For each item, the caregiver is asked to rate the frequency of the described behavior on a three-point Likert scale (0, not true; 1, somewhat or sometimes true; and 2, very true or often true). The checklist produces scores on seven quantitative syndrome scales (Emotionally Reactive, Anxious/Depressed, Somatic Complaints, Withdrawn, Sleep Problems, Attention Problems, and Aggressive Behavior), three broad-spectrum scales (Externalizing, Internalizing, and Total Problems), and five categorial DSM-oriented scales (Affective Problems, Anxiety Problems, Pervasive Developmental Problems, Attention Deficit/ Hyperactivity Problems, and Oppositional Defiant Problems). A T-score less than or equal to 63 for broad-spectrum scales, and 70 for syndromic and DSM-oriented scales is considered clinically significant; T-scores between 60 and 63 for broad-spectrum scales and between 65 and 70 for syndromic and DSM-oriented scales reveal a borderline level of symptoms; T-scores below 60 for broad-spectrum scales, and 65 for syndromic and DSM-oriented scales are considered normal [59].

For the purposes of our investigation, we assessed normal, borderline, or clinically relevant scores obtained by patients in the following scales: Emotionally Reactive, Aggressive Behavior, Anxiety Problems, Sleep Problems, Internalizing Problems, Externalizing Problems, and Total Problems.

#### 2.2.4. Sensory Processing

To obtain a detailed characterization of the participants’ sensory processing, a standardized and specifically developed questionnaire was administered to caregivers.

The SPM—Preschool Home Forms (SPM-P) is a parent-report measure of behaviors related to sensory processing, social participation, and praxis, specifically designed for children aged between 2 and 5 years. The SPM-P consists of 75 items, each rated on a four-point Likert scale, grouped into seven normative subscales, five of which investigate sensory system: vision (VIS), hearing (HEA), touch (TOU), body awareness (BOD), and balance and motion (BAL), with the last two subscales referring to proprioception and vestibular system, respectively. Two additional subscales—social participation (SOC) and planning and ideas (PLA)—concern higher integrative functioning—social functioning and praxis—and do not contribute to the total score. The total sensory system score (TOT) includes VIS, HEA, TOU, BOD, and BAL, as well as scores from a few additional items concerning taste and smell (TAS), which do not form a separate normative scale and for which normative scores are not available. SPM-P indices are T-scores, with a mean of 50 and a standard deviation of 10. T-scores between 40 and 59 are indicative of “typical functioning”, those between 60 and 69 are interpreted as “some problems”, and those between 70 and 80 are classified as “definite dysfunction”. T-scores interpreted as “some problems” and “definite dysfunction” should be considered atypical. In the Italian version of SPM-P, standardization yielded different T-scores in different age ranges (from 2 years to 2 years 11 months, and from 3 years to 5 years 11 months) [60].

### 2.3. Data Analysis

The Statistical Package for Social Sciences for Windows (SPSS) version 23.0 was used for all data analyses. Quantitative data are presented as mean ± standard deviation (SD). The Kolmogorov–Smirnov statistic assessed the normality of data on sensory processing, which showed a non-normal distribution of the investigated variables (test statistics ranging from 0.099 to 0.198; all *p* ≤ 0.01). Non-parametric tests were, therefore, chosen for statistical analysis. Pearson’s chi-squared test assessed sensory processing differences across gender and diagnosis. Fisher’s exact test was used instead of Pearson’s chi-squared test when the minimal sample size was small (more than 20% of outcomes with expected frequency < 5). Mann–Whitney U test was conducted to evaluate whether sensory processing differed by the presence of autistic core symptoms. Spearman’s rank correlation was computed to assess the relationship between sensory processing scores and developmental quotients and the relationship between sensory processing scores and behavioral problems scores.

## 3. Results

### 3.1. Sociodemographic Data, Developmental Level, Autistic Symptoms, and Behavioral Problems

The total sample included 141 children, aged between 23 months and 71 months (mean age: 41.5, with SD = 11.5), of whom 116 were male (82.3%) and 25 were female (17.7%). Among participants, 72 (51.1%) were diagnosed with ASD, while 69 (48.9%) received other diagnoses. In this group, which will be referred to as the clinical control group, 29 children (42.0%) were diagnosed with developmental delay, while 40 children (58.0%) were diagnosed with milder NDD (CD 56.4%, DCD 4.3%).

Developmental level, estimated either by a DQ or IQ, was grossly abnormal for 87 children (61.7%), mildly abnormal for 21 children (14.9%), and normal for 33 children (23.4%). The mean DQ in the total sample was 59.14. According to ADOS-2, moderate or severe autistic symptoms were found in 65 patients (46.1%). Table 1 summarizes these features.

ADI-R was administered to caregivers of 87 participants. For Scale A (Language and Communication), abnormal scores were obtained for 43 children (49.4%); for Scale B (Reciprocal Social Interactions), abnormal scores were reported in 52 cases (59.8%); and, for Scale C (Repetitive and Stereotyped Behaviors and Interests), abnormal scores were found for 42 participants (48.3%).

Among caregivers, 119 out of 146 completed the CBCL 1½–5 (60 ASD, and 59 other diagnoses). In the total sample, caregivers reported clinically significant behavioral problems in 26% of participants. The results are detailed in Table 2.

### 3.2. Sensory Processing

In the total sample, 85 children showed an SPM-P total T-score indicating typical functioning (60.3%), and 56 children had a total T-score interpreted as atypical (39.7%). Among the sensory subscales, participants most frequently showed atypical features in the vision subscale (40.4%), while the more rarely altered subscale was the body awareness subscale (26.2%). Atypical scores in at least one of the sensory domains investigated—vision, hearing, touch, body awareness, or balance and motion—were found in 88 participants (62.4%). The mean T-scores and frequencies of typical/atypical T-scores for all subscales and the total scale are reported in Table 3. For taste and smell items, the mean raw score is reported.

#### 3.2.1. Sensory Processing and Gender

In the total sample, males were found to have atypical scores more frequently than females in social participation, vision, hearing, and on the total scale. In contrast, females showed higher rates of atypical scores in all the other subscales. However, the gender differences were not statistically significant (all *p* > 0.05). The results are detailed in Table 4.

In the ASD group (59 males and 13 females), males more frequently had atypical scores than females in social participation, vision, hearing, balance and motion, planning and ideas, and the total scale; in contrast, females showed higher atypical scores in touch and body awareness. However, no statistically significant differences were found (all *p* > 0.05). The results are shown in Table 5.

In the clinical control group (57 males and 12 females), males were found to have atypical scores more frequently than females in the domains of social participation, touch, planning, ideas, and the total scale, while females showed higher rates of atypical scores in vision, hearing, body awareness, and balance and motion. However, no significant gender differences were highlighted (all *p* > 0.05). The results are detailed in Table 6.

#### 3.2.2. Differences in Sensory Processing Across Diagnoses

Comparing the frequencies of typical and atypical SPM-P scores between the two clinical groups, atypical scores were found more frequently in the ASD group. Statistically significant differences (*p* < 0.05) were found for the subscales “social participation” and “balance and motion”; a trend toward a statistically significant difference was also found in the subscale “body awareness” (*p* = 0.05). The results are shown in Table 7.

We found that 48 participants with ASD (66.7%) and 40 participants with other diagnoses (58%) had atypical scores in at least one of the investigated sensory domains—vision, hearing, touch, body awareness, or balance and motion.

#### 3.2.3. Sensory Processing and Autism Core Symptoms

For each ADI-R scale, we compared the SPM-P T-scores of patients with normal (lower than cut-off) and abnormal (equal to or higher than cut-off) scores for the considered scale. The mean raw scores were used for the taste and smell items.

The results for language and communication (Scale A) are reported in Table 8. Significant differences were found between the two groups for the SPM-P total score and for all subscales, except for “hearing” and “touch”, with patients with abnormal scores for Scale A showing greater SPM-P scores than those with normal scores.

For the Reciprocal Social Interactions Subscale (Scale B), significant differences were found between the two groups for the SPM-P total score and all subscales, except for “hearing” and “touch”, with patients with abnormal results for Scale B showing greater SPM-P scores than those with expected results. The results are detailed in Table 9.

Table 10 shows the results of the Repetitive and Stereotyped Behaviors and Interests scale (Scale C). Significant differences were found between the two groups for the SPM-P total score and all subscales, with patients with abnormal results for Scale C showing greater SPM-P scores than those with expected results.

#### 3.2.4. Sensory Processing and Level of Development

A developmental quotient was obtained for 130 patients (68 ASD, and 62 other diagnoses). We looked for a correlation between DQ and SPM-P T-scores. For taste and smell items, raw scores were used. In the total sample, a statistically significant negative correlation was found between DQ and scores on the subscales “social participation”, “taste and smell”, and “planning and ideas”. However, the strength of the correlations was weak.

In the ASD group, a statistically significant negative correlation was found between DQ and scores on the subscales “socialization”, “taste and smell”, “balance and motion”, and “planning and ideas”. The strength of the correlations was weak for the first three subscales and moderate for the last one.

No statistically significant correlations were found among participants with diagnoses other than ASD. Table 11 shows the results.

#### 3.2.5. Sensory Processing and Behavioral Problems

We looked for a correlation between SPM-P T-scores and behavioral problems, as measured by the CBCL ½–5 and classified as normal, borderline, or clinically relevant. For taste and smell items, raw scores were considered.

A strong correlation was found between the Total SPM-P T-score and the Total CBCL ½–5 score. Moreover, moderate correlations were found between most of the SPM-P subscales and CBCL ½–5 scores in the “Emotionally Reactive”, “Internalizing Problems”, and “Total Problems” subscales. The results are summarized in Table 12.

## 4. Discussion

The literature on atypical sensory processing in NDDs reports highly variable prevalence rates, ranging from 30% to 95% for ASD [14,61,62,63] and from 20% to 78% for other NDDs [9,63]. This is due to the high heterogeneity in the study methodology, clinical samples, and assessment tools, as well as the lack of consensus on the definition of a threshold for atypical sensory processing. Emblematic of this problem is the variety of terms used in the literature to indicate some form of atypical sensory processing: sensory processing disorder, sensory processing disorders, sensory processing issues, sensory differences, sensory modulation symptoms, sensory modulation disorders, sensory defensiveness, hypo- and hyper-responsivity, sensory symptoms, sensory processing dysfunctions, unusual sensory sensitivities, sensory anomalies, abnormal sensory responses, aberrant sensory features, multisensory dysfunctions, and multisensory deficits are just some examples. There is, however, unanimous agreement on the higher prevalence of sensory processing difficulties in children with NDDs when compared to TD peers, for whom the prevalence of atypical sensory processing is estimated to be between 3% and 16% [10,12].

In our study, the prevalence of globally atypical sensory processing—estimated by the Total SPM-P score—was 39.7%. The prevalence rate rose to 44.4% when considering only patients with a diagnosis of ASD. Among all the sensory domains, the one most frequently found impaired was vision, both in the total sample (40.4%) and in the ASD subgroup (44.4%), while the one least rarely impaired was proprioception—represented by the “body awareness” subscale—when the total sample was considered (26.0%), and hearing when only the ASD group was considered (27.8%).

No significant gender differences were found in the prevalence of atypical sensory processing among participants. The same results were observed when the prevalence of sensory issues was assessed separately for patients with ASD and children with other diagnoses. This result may be affected by the small number of females included in our sample, a common feature in clinical populations with NDDs and, even more, with ASD, where the male/female ratio has been reported to be as high as 7:1 [64]. Nevertheless, our findings align with those of some other studies on NDDs. In a clinical sample of children with ADHD and ASD, no significant gender differences were found in sensory processing issues [65]. Similar evidence comes from a more recent study on children and adolescents diagnosed with ASD, which reported the absence of significant gender differences in all the subscales of a parent-report questionnaire investigating sensory features [66]. In addition, some authors have recently proposed that sensory differences may be a sex-indifferent autism biomarker [67]. Evidence on this topic, however, is mixed, with conflicting results from different studies. Several authors have reported differences in how the ASD core symptomatology manifests in females and males [68,69], with boys showing more restricted and repetitive behaviors [70,71], a subset of which are atypical sensory features. A study on 195 children diagnosed with ASD reported significantly greater abnormalities in females for proprioceptive and vestibular impairments and for fears reflecting sensory avoidance but not for visual, auditory, olfactory, and tactile manifestations [72]. Other authors have found significant gender differences in auditory and vestibular abnormalities in a sample of ASD children, with females showing more severe symptoms [73]. Regarding other NDDs, one study found that females showed greater tactile hypersensitivity in a sample of ADHD children [74]. To the best of our knowledge, there is a lack of studies clarifying gender differences in sensory processing for clinical populations with DCD, CD, and developmental delay/ID. It seems that sex-based differences in sensory features in children with NDDs still need to be clarified; further research is needed, with larger samples and study designs considering other potentially confounding variables, such as age and cognitive functioning/developmental level [75].

The results of our study show a trend for children with ASD to have higher rates of atypical sensory processing in all sensory domains compared to peers with other neurodevelopmental diagnoses. However, it is interesting to note that the differences between the ASD group and the clinical control group reach statistical significance only in the social participation and vestibular domain—represented by the “balance and motion” subscale—and are close to statistical significance in the proprioceptive domain—represented by the “body awareness” subscale. The differences observed in social participation can easily be explained by the fact that social deficits are one of the key features of autism. Regarding proprioceptive and vestibular sensory inputs, it is well-known that ASD patients frequently show atypical features concerning the processing and integration of stimuli in these sensory systems [76,77,78,79]. To our knowledge, no research work focused on specifically comparing the vestibular and somatosensory processing of children in the autism spectrum with that of children with other NDDs. However, the available evidence on sensory differences across all modalities supports the existence of some differences between these clinical populations. Indeed, a previous study found that children with autism and an intellectual disability have qualitatively different sensory processing patterns than children with an intellectual disability alone [51], with ASD showing more atypical patterns in sensory sensitivity and sensory avoidance. Additionally, children with ASD have been found to exhibit more sensory symptoms than those with a developmental disability or language impairment [63]. Furthermore, recent research compared sensory modulation in children with ASD and with DCD, reporting that the former showed higher rates of atypical sensory patterns than the latter [80]. In contrast, studies comparing sensory processing between ASD and ADHD children have found no differences between these two populations [65,81]. It should be noted that, in our sample, the clinical control group included both children with DCD and children with developmental delay—a diagnosis that often precedes that of ID—and no ADHD children. The findings of our study, therefore, suggest that children in the autism spectrum are more affected by sensory processing difficulties than peers with other NDDs and that the proprioceptive and vestibular domains may be considered areas of specific vulnerability in the ASD population. This could explain the difficulties with movement co-ordination and postural control frequently encountered in clinical practice and research on children with ASD [82]. The evidence coming from our study may offer clinicians a red flag for investigating the presence of ASD in children with body awareness issues and balance difficulties. Moreover, these results could encourage the implementation of training in the domains of proprioception and balance in the context of rehabilitation interventions for children with ASD.

The results of our study show a significant association between atypical sensory processing and the presence of autism core symptoms reported by parents in the ADI-R. More specifically, all SPM-P subscales except “hearing” and “touch” are associated with language and communication deficits and reciprocal social interaction impairment. In contrast, all SPM-P subscales are associated with repetitive and stereotyped behaviors. This is in line with the evidence from the literature on the topic. Regarding social communication and interactions, a recent review focused on the neural and psychophysiological processes related to developing social communication behaviors in infants and toddlers [83]. Authors report that sensory processing, particularly of visual and auditory stimuli, is the first form of interaction that occurs between the infant and the social environment and, therefore, has a crucial role in the emergence of interactive behaviors and social communication, laying the foundations for high-level functions that appear throughout development. Regarding repetitive and stereotyped behaviors, it is well-known that one of the etiological explanations for motor stereotypes is that they act as arousal modulators when the person experiences sensory hypo- or hyper-stimulation [84]. A recent work of research offers new insights into how this may occur at a neural level. Authors suggest that the rhythmic brain signal generated to elicit the stereotyped movement and/or the rhythmic sensory feedback obtained as a result of the stereotypy entrains brain rhythms, disrupted in people with autism, to improve the processing of sensory information, often deficient in individuals with ASD [85]. Although our study does not establish a causal direction in the association between sensory processing difficulties and autism core symptoms, it is reasonable to hypothesize that sensory processing abnormalities, leading to abnormal arousal levels, distress, and emotional dysregulation, may further affect communication, social skills, and repetitive behaviors in children in whom these domains are already deficient due to their neurodevelopmental condition. Our findings are, therefore, of great interest because they support the possibility of reducing autism core symptoms with interventions specifically targeting sensory processing and with modifications of the environmental sensory features that disturb the child.

In the total sample of our study, only a weak negative correlation was found between developmental quotients and sensory abnormalities in the domain of “taste and smell”. In contrast, no correlation was found between development and the other sensory domains investigated. The results were similar when considering only the ASD patients, as well as when considering only the clinical control group. In the first case, we found only a weak negative correlation between DQ and scores of the subscales “taste and smell” and “balance and motion”; there was no correlation between the developmental quotient and abnormalities in the other sensory domains investigated. In the second case, no significant correlations were found. The absence of a relevant correlation between atypical sensory processing and developmental level found in our study is in line with the previous evidence found in the literature. Indeed, frequent abnormalities in sensory processing are also reported in individuals with high-functioning ASD [86,87], and, in a large sample of individuals with autism, the frequency of sensory symptoms did not differ significantly between low- and high-functioning subgroups [63]. In addition, atypical sensory processing is often reported in children with NDDs that do not cause impaired cognitive functioning, such as ADHD and DCD [9,47]. Moreover, results consistent with those of our study are reported by research on patients with different levels of intellectual disability, which found that the severity of ID has a minimal impact on sensory processing abilities and that all levels of ID show similar patterns of sensory impairment [41]. The results of our study should, therefore, encourage clinicians to investigate the presence of sensory processing difficulties even in patients with a normal or mildly abnormal level of development.

In our study, a positive correlation between sensory processing abnormalities and behavioral problems in children with NDDs was found. This is in line with the evidence in the literature on both neurotypical and neurodiverse children. A study including preschool-aged children with mixed neurodevelopmental conditions found a correlation between behavioral problems reported in the CBCL and sensory difficulties investigated through the Short Sensory Profile [88]. Furthermore, recent research on late preterm children reported a significant association between the occurrence of behavioral problems and atypical sensory processing at two years of age [89]. Similar findings come from studies on children with ASD, as well [90,91]. More specifically, in our sample, the strongest correlation between the SPM-P score and CBCL ½–5 score was found for the total scale of both questionnaires, and this may indicate a general, rather than domain-specific, correlation between atypical sensory processing and challenging behaviors, mediated by shared neuropsychological mechanisms. This agrees with recent findings suggesting a role for emotional dysregulation as a mediator between sensory processing and behavioral problems [90]. A significant but weak correlation was found between aggressive behavior and sensory difficulties. In the literature, aggressive behaviors are often reported to be associated with sensory processing dysfunctions in individuals with ASD, with studies mainly including adults, adolescents, or school-aged children [92,93,94,95]. This may highlight a tendency of younger children to express distress in non-aggressive rather than aggressive ways preferentially. However, it should be considered that caregivers of participants in our study may have underestimated aggressiveness given the very young age of children: this could be due to a perceived stigma associated with violent behaviors at such a young age or to the rare occurrence of injuries and damages caused by aggressive behavior when the preschool-aged child performs it. Regarding the CBCL ½–5 subscale “Anxiety problems”, it is noteworthy that there is a moderate correlation for this scale only for the total SPM-P score; in contrast, the correlation with the single sensory subscales is weak. This may indicate a cumulative effect of sensory processing impairments across multiple domains in generating anxiety, with anxiety symptoms more likely to be experienced by children who have sensory processing difficulties across multiple modalities. Finally, the lack of a correlation between CBCL ½–5 scores for “sleep problems” and any SPM-P subscale seems worthy of discussion. In our sample, the percentage of children who reported having at least some sleep problems on the CBCL ½–5 was 14.5%, a prevalence rate significantly lower than those reported in the literature for children with NDDs [96]. These findings appear to be determined by a low sensitivity of CBCL ½–5 questions, highlighting the presence of sleep disorders. Some authors have expressed concerns with the use of CBCL ½–5 to assess sleep since it is not a specifically designed questionnaire and does not have clear reference points for parents to assess some aspects of sleep, with responses to some items that could be affected by issues other than sleep in children, such as the child’s temperament [97]. The results of our study, therefore, indicate that sensory processing should be assessed in preschool children who present with behavioral problems. Furthermore, our work suggests the need to use tools specifically developed for sleep assessment to identify and treat sleep problems in preschool children with NDDs, given the crucial role of sleep at this developmental stage [98,99].

## 5. Conclusions

Atypical sensory processing is highly prevalent among children with neurodevelopmental conditions and can negatively impact their health, quality of life, and developmental trajectories. Our study shows that sensory processing difficulties can be detected even at a very young age. However, the assessment of sensory symptoms is not routinely included in diagnostic neuropsychiatric evaluation.

This study provides useful clinical and psychometric indicators that could guide clinicians in selecting patients at a higher risk for atypical sensory processing and who should be screened for this condition. Based on our findings, all children with a diagnosis of ASD or autism core symptoms should undergo a sensory processing assessment; gender and developmental level should not influence the decision to screen a child for atypical sensory processing.

Finally, we suggest investigating sensory processing in all children presenting problem behaviors. In conclusion, our results indicate that abnormalities in sensory processing should be considered to understand the clinical phenotypes of children with neurodevelopmental conditions in preschool age. Future research on larger samples will allow a better understanding of sensory processing features in this population, in order to implement targeted interventions in rehabilitation programs for these patients.

This study has some limitations that we would like to disclose. Firstly, we used parent-report measures to assess sensory processing, and, although the questionnaire we chose is well-validated, this could lead to potential biases. Future studies could consider implementing direct observations in the assessment of sensory processing. This work was also limited by different developmental levels among participants and the heterogeneity of the clinical control group (the “other diagnoses” group), which included children with very different neurodevelopmental conditions. Future research should be conducted to compare sensory processing between groups of children with homogeneous neurodevelopmental diagnoses at the same developmental level. Additionally, our sample was gender-biased, with a small number of females, which may have influenced the findings regarding gender differences. Given the male predominance of ASD and other NDDs, multicentric studies may be considered to obtain larger female samples.

Finally, the sample included in our research was not population-based but constituted of children brought to clinical attention. This may have led to a selection bias, with a higher rate of severely affected children and a lower number of individuals with milder symptoms included in the study. Future studies may consider selecting participants from the general population to obtain results that can be more reliably generalized to the entire clinical population of choice.

## Figures and Tables

**Table 1:** Demographic features, level of development, and autistic symptoms in total sample, ASD, and other diagnoses groups.

Characteristics	ASD (*n* = 72)	Other Diagnoses (*n* = 69)	Total Sample (*n* = 141)
Male (%)	59 (81.9%)	57 (82.6%)	116 (82.3%)
Female (%)	13 (18.1%)	12 (17.4%)	25 (17.7%)
Age (months: mean ± SD)	40.29 ± 10.65	42.70 ± 12.30	41,47 ± 11.51
Developmental quotient (mean ± SD)	49.44 ± 24.47	69.77 ± 23.94	59.14 ± 26.19
Grossly abnormal development (%)	56 (77.8%)	31 (44.9%)	87 (61.7%)
Mildly abnormal development (%)	6 (8.3%)	15 (21.7%)	21 (14.9%)
Normal development (%)	10 (13.9%)	23 (33.3%)	33 (23.4%)
Minimal or absent autistic symptoms (%)	0 (0%)	47 (68.1%)	47 (33.3%)
Mild autistic symptoms (%)	12 (16.7%)	17 (24.6%)	29 (20.6%)
Moderate or severe autistic symptoms (%)	60 (83.3%)	5 (7.2%)	65 (46.1%)

ASD = autistic spectrum disorder; SD = standard deviation.

**Table 2:** CBCL ½–5 results in total sample, ASD, and other diagnoses groups.

	ASD	Other Diagnoses	Total Sample
	(*n* = 69)	(*n* = 59)	(*n* = 119)
**Emotionally Reactive**			
Normal	46 (76.7%)	45 (76.3%)	91 (76.5%)
Borderline	6 (10%)	8 (13.6%)	14 (11.8%)
Clinical	8 (13.3%)	6 (10.2%)	14 (11.8%)
**Aggressive Behavior**			
Normal	50 (83.3%)	49 (83.1%)	99 (83.2%)
Borderline	8 (13.3%)	5 (8.5%)	13 (10.9%)
Clinical	2 (3.3%)	5 (8.5%)	7 (5.9%)
**Sleep Problems**			
Normal	55 (91.7%)	54 (91.5%)	109 (91.6%)
Borderline	0 (0%)	2 (3.4%)	2 (1.7%)
Clinical	5 (8.3%)	3 (5.1%)	8 (6.7%)
**Anxiety Problems**			
Normal	49 (81.7%)	49 (83.1%)	98 (82.4%)
Borderline	4 (6.7%)	3 (5.1%)	7 (5.9%)
Clinical	7 (11.7%)	7 (11.9%)	14 (11.8%)
**Internalizing Problems**			
Normal	32 (53.3%)	37 (62.7%)	69 (58%)
Borderline	10 (16.7%)	6 (10.2%)	16 (13.4%)
Clinical	18 (30%)	16 (27.1%)	34 (28.6%)
**Externalizing Problems**			
Normal	42 (70%)	44 (74.6%)	86 (72.3%)
Borderline	6 (10%)	4 (6.8%)	10 (8.4%)
Clinical	12 (20%)	11 (18.6%)	23 (19.3%)
**Total Problems**			
Normal	34 (56.7%)	43 (72.9%)	77 (64.7%)
Borderline	7 (11.7%)	4 (6.8%)	11 (9.2%)
Clinical	19 (31.7%)	12 (20.3%)	31 (26.1%)

ASD = autistic spectrum disorder.

**Table 3:** Mean SPM-P T-scores and frequencies of typical and atypical T-scores in the total sample.

	T-Score (mean ± SD)	Typical (%)	Atypical * (%)	Some Problems (%)	Definite Dysfunction (%)
Social participation	63.11 ± 13.36	56 (39.7%)	85 (60.3%)	30 (21.3%)	55 (39.7%)
Vision	57.93 ± 12.88	84 (59.6%)	57 (40.4%)	23 (16.3%)	34 (24.1%)
Hearing	53.26 ± 13.31	103 (73.0%)	38 (27.0%)	15 (10.6%)	23 (16.3%)
Touch	56.77 ± 13.13	85 (60.3%)	56 (39.7%)	28 (19.9%)	28 (19.9%)
Body awareness	53.79 ± 12.25	104 (73.8%)	37 (26.2%)	18 (12.8%)	19 (13.5%)
Balance and motion	54.01 ± 12.89	97 (68.8%)	44 (31.2%)	21 (14.9%)	23 (16.3%)
Planning and ideas	63.04 ± 13.53	60 (42.6%)	81 (57.4%)	27 (19.1%)	54 (38.3%)
Total	56.84 ± 12.87	85 (60.3%)	56 (39.7%)	31 (22.0%)	25 (17.7%)
Taste and smell, raw score	6.03 ± 2.47				

* Atypical includes “some problems” and “definite dysfunction”. SD = standard deviation.

**Table 4:** Typical and atypical SPM-P scores across gender in the total sample.

	Typical		Atypical		*p*
	Male, *n* (%)	Female, *n* (%)	Male, *n* (%)	Female, *n* (%)	
Social participation	43 (37.1%)	13 (52.0%)	73 (62.9%)	12 (48.0%)	0.166
Vision	69 (59.5%)	15 (60.0%)	47 (40.5%)	10 (40.0%)	0.962
Hearing	83 (71.6%)	20 (80.0%)	33 (28.4%)	5 (20.0%)	0.388
Touch	70 (60.3%)	15 (60.0%)	46 (39.7%)	10 (40.0%)	0.975
Body awareness	86 (74.1%)	18 (72.0%)	30 (25.9%)	7 (28.0%)	0.826
Balance and motion	80 (69.0%)	17 (68.0%)	36 (31.0%)	8 (32.0%)	0.925
Planning and ideas	50 (43.1%)	10 (40.0%)	66 (56.9%)	15 (60.0%)	0.776
Total	68 (58.6%)	17 (68.0%)	48 (41.4%)	8 (32.0%)	0.385

**Table 5:** Typical and atypical SPM-P scores across gender in the ASD group.

	Typical		Atypical		*p*
	Male, *n* (%)	Female, *n* (%)	Male, *n* (%)	Female, *n* (%)	
Social participation	13 (22.0%)	5 (38.5%)	46 (78.0%)	8 (61.5%)	0.289
Vision	31 (52.5%)	9 (69.2%)	28 (47.5%)	4 (30.8%)	0.273
Hearing	40 (67.8%)	12 (92.3%)	19 (32.2%)	1 (7.7%)	0.095
Touch	37 (62.7%)	6 (46.2%)	22 (37.3%)	7 (53.8%)	0.271
Body awareness	40 (67.8%)	8 (61.5%)	19 (32.2%)	5 (38.5%)	0.749
Balance and motion	35 (59.3%)	9 (69.2%)	24 (40.7%)	4 (30.8%)	0.507
Planning and ideas	21 (35.6%)	5 (38.5%)	38 (64.4%)	8 (61.5%)	1.000
Total	32 (54.2%)	8 (61.5%)	27 (45.8%)	5 (38.5%)	0.632

**Table 6:** Typical and atypical SPM-P scores across gender in clinical control group.

	Typical		Atypical		*p*
	Male, *n* (%)	Female, *n* (%)	Male, *n* (%)	Female, *n* (%)	
Social participation	30 (52.6%)	8 (66.7%)	27 (47.4%)	4 (33.3%)	0.374
Vision	38 (66.7%)	6 (50.0%)	19 (33.3%)	6 (50.0%)	0.330
Hearing	43 (75.4%)	8 (66.7%)	14 (24.6%)	4 (33.3%)	0.497
Touch	33 (57.9%)	9 (75.0%)	24 (42.1%)	3 (25.0%)	0.342
Body awareness	46 (80.7%)	10 (83.3%)	11 (19.3%)	2 (16.7%)	1.000
Balance and motion	45 (78.9%)	8 (66.7%)	12 (21.1%)	4 (33.3%)	0.453
Planning and ideas	29 (50.9%)	5 (41.7%)	28 (49.1%)	7 (58.3%)	0.562
Total	36 (63.2%)	9 (75.0%)	21 (36.8%)	3 (25.0%)	0.521

**Table 7:** Comparison of typical and atypical SPM-P scores between ASD and clinical control group.

	Typical		Atypical		*p*
	ASD, *n* (%)	Other Diagnoses, *n* (%)	ASD	Other Diagnoses, *n* (%)	
Social participation	18 (25%)	38 (55.1%)	54 (75%)	31 (44.9%)	<0.001 *
Vision	40 (55.6%)	44 (63.8%)	32 (44.4%)	25 (36.2%)	0.321
Hearing	52 (72.2%)	51 (73.9%)	20 (27.8%)	18 (26.1%)	0.821
Touch	43 (59.7%)	42 (60.9%)	29 (40.3%)	27 (39.1%)	0.889
Body awareness	48 (66.7%)	56 (81.2%)	24 (33.3%)	13 (18.8%)	0.051
Balance and motion	44 (61.1%)	53 (76.8%)	28 (38.9%)	16 (23.2%)	0.044 *
Planning and ideas	26 (36.1%)	34 (49.3%)	46 (63.9%)	35 (50.7%)	0.114
Total	40 (55.6%)	45 (65.2%)	32 (44.4%)	24 (34.8%)	0.241

* *p* < 0.05. ASD = autistic spectrum disorder.

**Table 8:** Differences in SPM-P scores between patients with and without “Language and Communication” abnormalities reported at the ADI-R.

SPM-P	ADI-R (A)	Mean Rank	z	*p*
**Social participation, T-score**	Abnormal	54.79	3.963	<0.001 *
	Normal	33.45		
**Vision, T-score**	Abnormal	53.80	3.584	<0.001 *
	Normal	34.42		
**Hearing, T-score**	Abnormal	49.31	1.951	0.051
	Normal	38.81		
**Touch, T-score**	Abnormal	47.59	1.315	0.189
	Normal	40.49		
**Taste and smell, raw score**	Abnormal	53.93	3.708	<0.001 *
	Normal	34.30		
**Body awareness, T-score**	Abnormal	51.23	2.652	0.008 *
	Normal	36.93		
**Balance and motion, T-score**	Abnormal	57.00	4.763	<0.001 *
	Normal	31.30		
**Planning and ideas, T-score**	Abnormal	53.91	3.638	<0.001 *
	Normal	34.32		
**Total, T-score**	Abnormal	53.95	3.641	<0.001 *
	Normal	34.27		

* *p* < 0.05.

**Table 9:** Differences in SPM-P scores between patients with and without “Reciprocal Social Interactions” abnormalities reported at the ADI-R.

SPM-P	ADI-R (B)	Mean Rank	z	*p*
**Social participation, T-score**	Abnormal	50.70	3.035	0.002 *
	Normal	34.04		
**Vision, T-score**	Abnormal	48.46	2.012	0.044 *
	Normal	37.37		
**Hearing, T-score**	Abnormal	45.99	0.901	0.368
	Normal	41.04		
**Touch, T-score**	Abnormal	46.66	1.202	0.229
	Normal	40.04		
**Taste and smell, raw score**	Abnormal	49.58	2.568	0.010 *
	Normal	35.71		
**Body awareness, T-score**	Abnormal	48.94	2.235	0.025 *
	Normal	36.66		
**Balance and motion, T-score**	Abnormal	50.48	2.928	0.003 *
	Normal	34.37		
**Planning and ideas, T-score**	Abnormal	50.91	3.130	0.002 *
	Normal	33.73		
**Total, T-score**	Abnormal	49.05	2.277	0.023 *
	Normal	36.50		

* *p* < 0.05.

**Table 10:** Differences in SPM-P scores between patients with and without “Repetitive and Stereotyped Behaviors and Interests” abnormalities reported at the ADI-R.

SPM-P	ADI-R (B)	Mean Rank	z	*p*
**Social participation, T-score**	Abnormal	53.95	3.572	<0.001 *
	Normal	34.71		
**Vision, T-score**	Abnormal	54.63	3.799	<0.001 *
	Normal	34.08		
**Hearing, T-score**	Abnormal	53.11	3.267	0.001 *
	Normal	35.50		
**Touch, T-score**	Abnormal	51.52	2.691	0.007 *
	Normal	36.98		
**Taste and smell, raw score**	Abnormal	51.73	2.820	0.005 *
	Normal	36.79		
**Body awareness, T-score**	Abnormal	53.40	3.371	0.001 *
	Normal	35.22		
**Balance and motion, T-score**	Abnormal	55.15	3.994	<0.001 *
	Normal	33.59		
**Planning and ideas, T-score**	Abnormal	53.05	3.247	0.001 *
	Normal	35.56		
**Total, T-score**	Abnormal	56.04	4.303	<0.001 *
	Normal	32.77		

* *p* < 0.05.

**Table 11:** Correlations between SPM-P scores and developmental quotients in total sample, ASD, and clinical control groups.

		Developmental Quotient		
		Total Sample	ASD	Other Diagnoses
**Social participation, T-score**	Correlation Coefficient	−0.337 **	−0.302 *	−0.108
	Sig. (2-tailed)	0.000	0.012	0.402
**Vision, T-score**	Correlation Coefficient	−0.197 *	−0.198	−0.181
	Sig. (2-tailed)	0.024	0.105	0.160
**Hearing, T-score**	Correlation Coefficient	−0.023	0.021	−0.062
	Sig. (2-tailed)	0.797	0.866	0.633
**Touch, T-score**	Correlation Coefficient	−0.119	−0.213	−0.012
	Sig. (2-tailed)	0.178	0.082	0.927
**Taste and smell, raw score**	Correlation Coefficient	−0.242 **	−0.287 *	−0.152
	Sig. (2-tailed)	0.006	0.018	0.239
**Body awareness, T-score**	Correlation Coefficient	−0.059	−0.140	0.078
	Sig. (2-tailed)	0.502	0.254	0.574
**Balance and motion, T-score**	Correlation Coefficient	−0.119	−0.308 *	0.109
	Sig. (2-tailed)	0.179	0.011	0.400
**Planning and ideas, T-score**	Correlation Coefficient	−0.329 **	−0.488 **	−0.174
	Sig. (2-tailed)	0.000	0.000	0.176
**Total, T-score**	Correlation Coefficient	−0.170	−0.227	0.086
	Sig. (2-tailed)	0.053	0.063	0.506

* Correlation is significant at the 0.05 level; ** Correlation is significant at the 0.01 level. ASD = autistic spectrum disorder.

**Table 12:** Correlations between SPM-P scores and CBCL ½–5 results.

		SOC	VIS	HEA	TOU	TAS	BOD	BAL	PLA	TOT
**Emotionally reactive**	Correlation Coefficient	0.322 **	0.450 **	0.426 **	0.423 **	0.369 **	0.472 **	0.427 **	0.465 **	0.505 **
	Sig. (2-tailed)	0.000	**0.000**	**0.000**	**0.000**	0.000	**0.000**	**0.000**	**0.000**	**0.000**
**Aggressive behavior**	Correlation Coefficient	0.308 **	0.284 **	0.305 **	0.339 **	0.189*	0.321 **	0.315 **	0.339 **	0.360 **
	Sig. (2-tailed)	0.001	0.002	0.001	0.000	0.039	0.000	0.000	0.000	0.000
**Anxiety problems**	Correlation Coefficient	0.335 **	0.307 **	0.335 **	0.358 **	0.262 **	0.379 **	0.351 **	0.336 **	0.403 **
	Sig. (2-tailed)	0.000	0.001	0.000	0.000	0.004	0.000	0.000	0.000	**0.000**
**Sleep problems**	Correlation Coefficient	0.115	0.164	0.223*	0.205 *	0.182	0.230 *	0.151	0.151	0.256 **
	Sig. (2-tailed)	0.211	0.074	0.015	0.025	0.047	0.012	0.100	0.101	0.005
**Internalizing problems**	Correlation Coefficient	0.433 **	0.451 **	0.482 **	0.483 **	0.389 **	0.487 **	0.488 **	0.484 **	0.574 **
	Sig. (2-tailed)	**0.000**	**0.000**	**0.000**	**0.000**	0.000	**0.000**	**0.000**	**0.000**	**0.000**
**Externalizing problems**	Correlation Coefficient	0.329 **	0.384 **	0.320 **	0.403 **	0.328 **	0.390 **	0.463 **	0.406 **	0.457 **
	Sig. (2-tailed)	0.000	0.000	0.000	**0.000**	0.000	0.000	**0.000**	**0.000**	**0.000**
**Total problems**	Correlation Coefficient	0.422 **	0.485 **	0.419 **	0.549 **	0.438 **	0.542 **	0.561 **	0.556 **	0.618 **
	Sig. (2-tailed)	**0.000**	**0.000**	**0.000**	**0.000**	**0.000**	**0.000**	**0.000**	**0.000**	**0.000**

* Correlation is significant at the 0.05 level; ** Correlation is significant at the 0.01 level. Moderate correlations in bold; strong correlations in bold underlined. SOC = social participation; VIS = vision; HEA = hearing; TOU = touch; TAS = taste and smell; BOD = body awareness; BAL = balance and motion; PLA = planning and ideas; TOT = total sensory system score.

## Data Availability

The data presented in this study are available upon request from the corresponding author. The data are not publicly available due to privacy.
